# From Systemic Inflammation to Neuroinflammation: The Case of Neurolupus

**DOI:** 10.3390/ijms19113588

**Published:** 2018-11-13

**Authors:** Mykolas Bendorius, Chrystelle Po, Sylviane Muller, Hélène Jeltsch-David

**Affiliations:** 1 UMR 7242 Biotechnologie et Signalisation Cellulaire, École Supérieure de Biotechnologie de Strasbourg (ESBS), Laboratoire d’Excellence Médalis, Université de Strasbourg/CNRS, 67412 Illkirch, France; mbendorius@unistra.fr (M.B.); sylviane.muller@unistra.fr (S.M.); 2 ICube UMR 7357, Université de Strasbourg/CNRS, Fédération de Médecine Translationnelle de Strasbourg, 67000 Strasbourg, France; chrystelle.po@unistra.fr; 3 University of Strasbourg Institute for Advanced Study (USIAS), 67000 Strasbourg, France

**Keywords:** autoimmunity, systemic lupus erythematosus (SLE), neuropsychiatric lupus (NPSLE), murine model, magnetic resonance imaging (MRI), blood–brain barrier, behavior

## Abstract

It took decades to arrive at the general consensus dismissing the notion that the immune system is independent of the central nervous system. In the case of uncontrolled systemic inflammation, the relationship between the two systems is thrown off balance and results in cognitive and emotional impairment. It is specifically true for autoimmune pathologies where the central nervous system is affected as a result of systemic inflammation. Along with boosting circulating cytokine levels, systemic inflammation can lead to aberrant brain-resident immune cell activation, leakage of the blood–brain barrier, and the production of circulating antibodies that cross-react with brain antigens. One of the most disabling autoimmune pathologies known to have an effect on the central nervous system secondary to the systemic disease is systemic lupus erythematosus. Its neuropsychiatric expression has been extensively studied in lupus-like disease murine models that develop an autoimmunity-associated behavioral syndrome. These models are very useful for studying how the peripheral immune system and systemic inflammation can influence brain functions. In this review, we summarize the experimental data reported on murine models developing autoimmune diseases and systemic inflammation, and we explore the underlying mechanisms explaining how systemic inflammation can result in behavioral deficits, with a special focus on in vivo neuroimaging techniques.

## 1. Introduction

The effects of systemic inflammation on the central nervous system (CNS) are quite well illustrated in neuropsychiatric systemic lupus erythematosus (NPSLE), a poorly understood, severe form of systemic lupus erythematosus (SLE) disease that can affect up to 75% of SLE patients. This dramatic form of lupus disease covers a wide range of manifestations that are divided into focal and diffuse ones. Focal symptoms (e.g., seizures, cerebrovascular disease, aseptic meningitis) usually result from a stroke occurring in a specific structure and can be readily detected by magnetic resonance imaging (MRI), while diffuse symptoms (e.g., depression, cognitive dysfunction, mood and anxiety disorders, acute confusional state, psychosis), however, are much harder to identify accurately by MRI and can have debilitating consequences for patients as well. It is thought that diffuse symptoms proceed due to neuroinflammatory processes both in the periphery and the CNS, which we seek to explore in this review. After summarizing the main features of the pathology and the mouse models that recapitulate the human disease in terms of peripheral inflammation and behavioral deficiencies, we inquire into how advanced MRI techniques can be used non-invasively in mice to identify NPSLE symptoms and how this might translate to the human disease. At last, the way in which neuroinflammation may cause central nervous symptoms in these murine lupus-prone models is explored.

## 2. Systemic and NP Aspects of Lupus Disease

### 2.1. General Presentation of SLE

SLE is a chronic relapsing-remitting autoimmune disease characterized by a rupture of self-tolerance and systemic inflammation mainly resulting from the hyperactivation of peripheral B and T cells [1], resulting in high levels of pathogenic autoantibodies (autoAbs), tissue deposition of immune complexes, and, ultimately, multiple and various organ injuries (e.g., skin, kidneys, heart, lungs, brain) [2,3,4]. The disease primarily affects females of childbearing age (90% of patients) [5], and its etiology, which appears multigenic, is not fully understood due to it being also influenced by hormonal and environmental factors (e.g., UV radiation, diet, smoke, infections, pollutants, stress) [6,7,8].

### 2.2. CNS Involvement in Human SLE: The Neuropsychiatric Lupus Disease (NPSLE)

Depending on the study, a varying number of SLE sufferers (from 15% to 75%) present with neuropsychiatric (NP) symptoms that cover the whole spectrum of psychiatric dysfunction [9,10]. These NP manifestations are associated with a reduced quality of life [11,12,13] and, when severe, they substantially contribute to the morbidity and mortality rates of SLE patients [14]. Nowadays, the etiology of NPSLE remains poorly understood, as is also the case for SLE and most autoimmune diseases.

In 1999, the American College of Rheumatology (ACR) recognized 19 wide-ranging NP manifestations related to SLE [15]: some affect the CNS, in which case they can be focal or diffuse [16], and the others affect the peripheral nervous system (Table 1). This widely adopted classification was meaningful from a clinical point of view but presents some limitations with respect to the variety of symptoms and their specific attribution to SLE [11]. In 2001, Ainiala et al. revised this original categorization and discarded certain minor NP symptoms, such as headaches and anxiety disorders [17,18]. When compiling recently published data, it clearly appears that this novel classification has affected the NPSLE prevalence values, pointing to the lack of consensus on straightforward and uncontroversial NPSLE diagnosis (e.g., definition of impairment, selection of patients and of cognitive tests used). Since then, various NPSLE diagnostic criteria have been proposed but none of them have achieved both high sensitivity and specificity [19]. In fine, there is currently no consensus about inclusion or exclusion criteria for NPSLE, resulting commonly in an overdiagnosis of NPSLE and the administration of unnecessary immunosuppressive treatments.

One critical problem with the diagnosis of NPSLE remains the lack of objective and specific biomarkers. Therefore, this aspect remains eminently challenging. Today, NPSLE is essentially clinically defined by physical examination, serological measures, psychological and neurological evaluations, and brain imaging. Hopefully, rapidly advancing progress in live imaging technology will help and allow the establishment of reliable and specific diagnostic criteria for NPSLE.

Twenty autoAbs (11 brain-specific and 9 systemic) [20] and several cytokines found in the serum, but more specifically in the cerebrospinal fluid (CSF) of patients, have been linked to NPSLE [10]. Focal manifestations can be detected by MRI techniques and reflect, usually, cerebral vasculopathy, thrombosis, and complement activation [10,11,16]. The presence of antiphospholipid (aPL) autoAbs in the CSF correlates with some of these events [16]. Diffuse symptoms are harder to identify [21] and seem related, instead, to inflammation elicited by several mediators (e.g., interferon (IFN)α, anti-N-methyl-d-aspartate receptor (NMDAR and anti-ribosomal P autoAbs), which induce not only leakage of the blood–brain barrier (BBB) but also other barriers, as briefly addressed below [22,23,24,25,26,27].

The brain is protected from the periphery by three distinct structural and functional interfaces with systemic circulation: the BBB, the blood–CSF barrier (BCSFB), and the meningeal barrier [28]. These entities comprise endothelial cells (ECs) connected by tight junctions that prevent free passage of soluble macromolecules and cells, control the influx of nutrients and efflux of toxic molecules, and maintain a regulated microenvironment optimal for neuronal signaling [29,30]. The BBB has been proposed to be leaky both in SLE patients and lupus-prone mice [31], resulting in the deleterious diffusion of proinflammatory factors. In humans, this alteration has been evidenced mainly by the presence of serum albumin and immunoglobulins G (IgGs) in the CSF [26,32,33] and through the use of MRI [34]. Previously, it was claimed that BBB disruption was directly responsible for the passage of peripheral molecules to the brain, initiating NPSLE disease. However, recent data obtained from experiments with mice argue that the peripheral molecules reach the brain through the BCSFB rather than via the BBB [22], providing interesting hypotheses in favor of BCSFB leakage in human patients as well [35], and refining the BBB leakage dogma.

### 2.3. Modelization of the Disease in Mice

There are obvious limits to the search for mechanisms of CNS disease in human patients. Thus, murine models offer several advantages for elucidating the early mechanisms of NP manifestations of SLE and help to distinguish between CNS-specific and -nonspecific mechanisms [36]. Neuroinflammatory mechanisms of NPSLE are quite well recapitulated in lupus-prone murine models that develop a lupus-like disease [37], including NP events, manifesting through the production of autoAbs in the serum [38] and CSF [39,40]. Some animal models are spontaneous, as the BXSB mice, the Murphy Roths Large (MRL) mice, and the F1 hybrid of the New Zealand Black (NZB) and New Zealand White (NZW) mouse strain (called (NZB × NZW) F1) [41]. Others are genetically engineered, such as the 564Igi mouse [42,43]. All these murine models help to provide valuable insights into how the CNS can be affected by systemic inflammation. Nevertheless, after the identification of autoAbs targeting the brain, NPLSE-like inducible NPSLE models have also been developed. In these induced models, human autoAbs are passively injected or, alternatively, mice are immunized against these specific brain self-antigens, leading to the development of NPLSE-like disease without the severe peripheral autoimmune damage [44,45,46,47]. Even if none of these animal models reflect the human disease perfectly, they all provide some key elements involved in the disease’s pathogenesis, leading to the development of safer and more efficient treatments, as was very well depicted in a recent review [48].

The MRL/lpr strain is one of the best-established spontaneous models of SLE and is the most commonly investigated in lupus-related NP studies. This strain displays an autoimmune phenotype, and its composite genome is derived from LG/J (75%), AKR/J (12.6%), C3H/HeDi (12.1%), and C57BL/6J (0.3%) mice [49]. MRL/lpr mice spontaneously develop an autosomal recessive lymphoproliferation (lpr) mutation affecting the *Fas* gene [50]. Identified as an intron deletion in the *Fas* gene, this mutation leads to aberrant *Fas* mRNA splicing [50] and the loss of protein expression [51]. Interestingly, this mutation does not necessarily increase the proliferative capacity of lymphocytes but rather allows them to escape the negative and positive selection processes [50,51]. The parental strain of MRL/lpr mice, the MRL/MpJ mouse (also named MRL^+/+^ later in the text), does not carry the lpr mutation and develops the autoimmune syndrome, albeit weaker and delayed in life, thus representing a natural and adequate control [52]. Over the past decade, the MRL/lpr exhibited a “fortuitous attenuation” of symptoms of still unknown origin, and the MRL/lpr original stock was re-established in 2008 (http://jaxmice.jax.org/strain/006825.html) [36,41].

**Figure 1:**
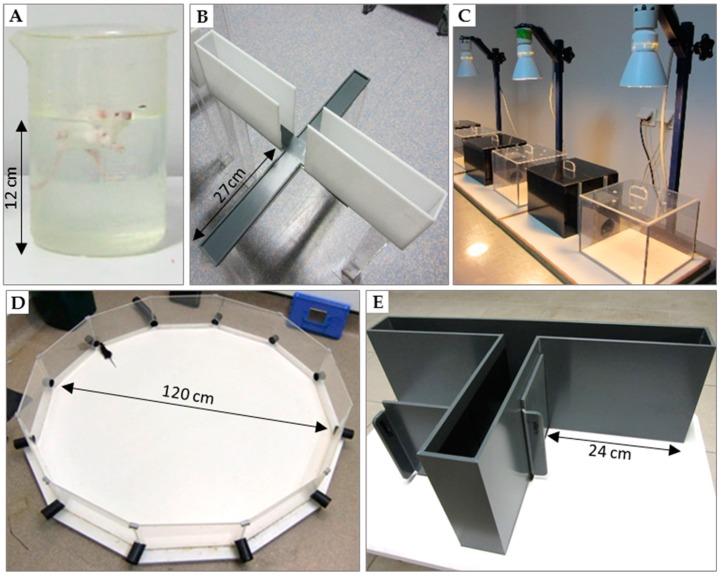
Tests used for behavioral evaluation of mice. (**A**) Forced-swim test (or Porsolt test) used to assess depressive-like behavior; (**B**) elevated plus-maze and (**C**) dark/light preference test to measure anxiety-like behavior; (**D**) Morris water maze (or paddling test) [53]; (**E**) T-maze that is considered very sensitive to alteration in hippocampal pathways.

The disease developed by MRL/lpr mice mimics human SLE. In particular, in both settings, a leaky BBB and the presence of circulating autoAbs directed against dsDNA and Smith (Sm) antigen are observed, the only serological biomarkers of SLE in humans. As in human SLE, where a strong gender preference is usually described (9:1 female to male ratio), female MRL/lpr mice develop a more severe disease [41]. Finally, comparable to some NP symptoms evidenced in SLE patients, autoimmune MRL/lpr mice spontaneously develop pathological changes in the brain and an autoimmunity-associated behavioral syndrome (e.g., depression, emotional and cognitive dysfunction) that can be characterized with a battery of behavioral tests, summarized below (Figure 1). However, it is important to emphasize that, in this murine model, the disease-associated brain atrophy remains poorly understood.

#### 2.3.1. Tests of Depression

• Forced-swim test (Porsolt test; Figure 1A)

In this experimental procedure, a mouse is forced to swim in a small container without the possibility to escape. Quickly, after a time of struggling and swimming, the animal stops any attempt of escape and becomes almost motionless (e.g., floating on the water surface and moving its limbs only to rebalance itself). Periods of activity alternate with periods of immobility, and the duration of the latter increases with time. Within this framework, immobility objectivizes behavioral helplessness and is interpreted as behavioral “distress” that ensues when the animal realizes that it cannot escape [54]. This model of behavioral despair is repeatedly validated by the pharmacological plan to detect the efficacy of antidepressants. The quicker the animal becomes immobile and stops trying to escape, i.e., the longer it stays immobile, the greater extent to which it is thought to display depressive symptoms. As compared to their parental MRL^+/+^ littermates used as controls, MRL/lpr mice exhibit defects [55,56,57] implying an autoimmunity-associated syndrome.

• Sucrose preference test

This test evaluates deficiencies in motivated and goal-directed behavior and anhedonia, i.e., a reduced sensitivity to positive stimuli. Here, MRL/lpr mice manifest a reduced preference for palatable drinking solutions with sucrose compared to control MRL^+/+^ mice. This observation is well established and can be detected both early in life and later during disease progression [57,58,59,60,61].

• Open-field locomotor activity test

Depressive-like manifestations also include apathy and fatigue, which are reflected as a decrease in spontaneous exploration of a novel environment, like an open field. Generally, as compared to control mice, MRL/lpr mice show such a decrease in spontaneous locomotion [55,56,62].

#### 2.3.2. Anxiety Tests

• Elevated plus-maze and dark/light preference tests (Figure 1B,C)

The elevated plus-maze and the dark/light preference tests are usually implemented in pharmacology for the screening of anxiolytic molecules [63,64,65]. The plus-maze test consists of a cross structure with two arms with walls (closed arms) and two wall-free arms (open arms). The dark/light preference test consists of two boxes, one is dark and the other is well lit, and an opaque tunnel that connects the dark box with the lit one. Both tests create an approach-avoidance conflict between the natural tendency of mice to explore and their aversion to open or brightly lit spaces. Generally, the more anxious the animal is, the less it will venture and stay in the open or lit compartments. Some debatable results have been reported in MRL/lpr mice with the elevated plus-maze test. Sakić and colleagues observed anxiety in MRL/lpr mice, i.e., diminution of the time spent in the open arms [55], while other groups noted the opposite result, with MRL/lpr mice being significantly less anxious than their MRL^+/+^ littermates [57,66,67]. An important experimental difference between these two sets of studies is that some experiments were conducted with males [55] and others with females [57,66,67].

• Open-field test

The open-field test is sometimes used to measure anxiety-related behavior, as quantified by the duration for which the mouse avoids the central part of a novel enclosed arena and remains in close proximity of the walls (thigmotaxis) [68,69]. In this test, as compared to control MRL^+/+^ mice, MRL/lpr mice disclose anxiety-like behavior, as evidenced by increased thigmotaxis and impaired exploration of space [55,57].

Controversial results have been reported for open-field and elevated plus-maze tests that might be explained by the use of different strains and sex of mice [70,71]. To discuss the absence of consensus in detail is beyond the scope of this essay but, as well depicted in an exhaustive review, it is worth mentioning that independent of the strain, male and female mice display inherently different anxiety-coping mechanisms [72]. Potential processes underlying sex differences in anxiety states include emerging evidence supporting the existence of two anatomically and functionally distinct serotonergic circuits that modulate conflict anxiety and panic-like anxiety, respectively. Regarding the MRL/lpr strain, female MRL/lpr mice seem to display, as in human SLE, a strong bias in severity (e.g., higher levels of IgG in the CSF) [40] and rate of progression of the autoimmune disease (e.g., earlier apparition of serum autoAbs) [57], which manifest in stronger cerebral pathology [56,66,73]. Therefore, sex differences in the expression of anxiety behavior are not surprising or unexpected in MRL/lpr mice.

#### 2.3.3. Cognitive Tests

The detection of cognitive deficits in animals seems to be task-dependent. This finding is hardly surprising with regard to the fact that there are multiple “types” of memory, which are differently sensitive to brain damages [74]. Even if the three tests listed below are classified as “cognitive” tasks, they do not measure the same “cognitive processes” (working (or short-term) visual memory in the novel object recognition test [75], spatial working and reference (or long-term) memory in the Morris water maze, spatial working memory in the T-maze that is also sensitive to nonspatial learning aspects (e.g., temporal discriminations) [76]). Furthermore, these tests differ in terms of perceptual stimuli cueing choice behavior, and their accomplishments rely on different mechanisms, some being noncognitive (e.g., motivational factors), subserved by the activity of many brain regions.

• Novel object recognition test

This test is based on the tendency of rodents to preferentially explore novel objects. First, a mouse is placed into an arena and allowed to explore two identical novel objects. After a fixed period of exploration (defined as rearing on the object, whisking, sniffing, touching with nose and/or forepaws), the mouse is removed. Next, one of the objects is changed to a novel object and the mouse is reintroduced into the arena. Mice naturally tend to explore novel objects, so the duration of exploring the novel object is taken as the measure of visual working memory. In this paradigm of testing, MRL/lpr mice perform as well as MRL^+/+^ controls [66] or even outperform them [57].

• Morris water maze (Figure 1D)

The Morris water maze test evaluates spatial memory in rodents [77]. The animal is placed into a tank filled with opaque water in which a platform is hidden beneath the surface. In this aversive situation, mice must learn using spatial cues placed in the testing room to navigate and find the platform. Longer latencies to find the platform reflect poorer performances. In this test, MRL/lpr mice show pronounced thigmotaxic swimming but no clear-cut impairment in learning/memory abilities compared to MRL^+/+^ littermates [56,78,79].

• The T-maze alternation test (Figure 1E)

This test, shaped like the letter T, is based on the willingness of rodents to explore a new environment, i.e., they prefer to visit a new arm of the maze rather than the familiar one. The solving of this task consists of two turns, i.e., right and left. Mice are first placed in the start arm of the T-maze. Upon leaving the start arm, they choose between entering either the left or the right goal arm. With repeated trials, the animals show less tendency to enter a previously visited arm. The percentage of alternation (number of turns in each goal arm) is recorded. This test is currently used to evaluate cognitive deficits in mice and test novel chemical entities for their effects on cognition. The T-maze test is well known as particularly sensitive for detecting hippocampal dysfunction [80,81,82,83]. In MRL/lpr mice, alternation impairments have been evidenced as compared to the MRL^+/+^ counterparts [84,85], strongly supporting cognitive and, more specifically, hippocampal dysfunction in these mice.

#### 2.3.4. Locomotor function

• Beam-walking test

The beam-walking test assesses the capacity of mice to coordinate movement on a narrow beam [67]. Mice are placed on the beam and the latency to cross and the number of paw slips are recorded. Interestingly, female MRL/lpr mice do not display locomotor deficits compared to their control MRL^+/+^ littermates [49,50]. However, males perform significantly worse [50]. The precise mechanisms of this sex difference remain unclear but might be due to different hormonal backgrounds [86].

Other tests assessing locomotor functions have been described, such as the pole test and string agility test, for example [68,69]. These tests, which require a good driving agility, give an idea of the integrity of proprioceptive and vestibular pathways. Generally, they are used in order to quantify the effects of either alterations in the CNS (motor cortex), those of various neuromuscular pathologies, or those of fatigue on the fine motricity and proprioceptive functions. These tests also allow checking for the presence of possible sensory-motor biases, which could affect observations obtained in other tests.

The characterization of specific inflammatory factors giving rise to an autoimmunity-associated behavioral syndrome has contributed to the development of NPSLE inducible murine models. For example, some NP features have been observed in C3H/HeJ mice after ribonucleoprotein P human autoAb intracerebroventricular injection [87]. Anti-NMDAR Abs effects have been studied following injection of human anti-dsDNA Abs into the hippocampi of C57BL/6 mice [46] or immunization of BALB/c mice with the DWEYS peptide [44,45,88,89]. To date, only a few inducible models have been yet developed, but it is anticipated that a growing number of relatively specific models of NPSLE will be generated in the nearest future. Taken together, we can conclude that spontaneous and inducible NPSLE models fairly well recapitulate the human disease and provide important insights into how systemic inflammation can induce CNS damage. This central aspect is discussed below.

## 3. Neuroimaging in NPSLE

### 3.1. Magnetic Resonance Imaging Modalities

As already mentioned, two distinct, but potentially complementary, pathogenic mechanisms are distinguished in NPSLE: (i) vascular/thrombotic injury, likely implicating aPL Abs; and (ii) inflammation-mediated injury, implicating other pathogenic autoAbs, proinflammatory cytokines (e.g., interleukin (IL)-6, IFNα), and disruption of the BBB, the latter being favored, among other factors, by the activation of the complement system and binding of immune complexes to ECs [90,91]. Nowadays, implementation of noninvasive anatomical and functional neuroimaging modalities is highly recommended for the identification of outcomes of pathogenic mechanisms that impact the structure, metabolism, and functionality of the brain.

MRI detects signals thanks to the properties of hydrogen nuclei in water and fat molecules, which are found in biological tissues, including the brain. It is the most relevant neuroimaging technique for the detection of structural alterations in the CNS (e.g., cortical atrophy, focal periventricular and subcortical white matter lesions, diffuse gray matter changes, reduced volume of the corpus callosum or the hippocampus) [92]. It is thus particularly sensitive to detecting acute focal NP manifestations [93,94,95,96,97,98,99,100,101]. Unfortunately, its diagnostic value remains limited since, in the case of diffuse manifestations, MRI usually gives unremarkable results or shows nonspecific abnormalities. To make matters worse, cerebral MRI abnormalities can be observed in SLE patients without NP symptoms and are even sometimes detected in healthy individuals [102,103,104].

Today, several advanced imaging modalities exist. These include diffusion-weighted imaging (DWI) [105], magnetic transfer imaging (MTI) [106], magnetic resonance angiography (MRA) [107], magnetic resonance spectroscopy (MRS) [108], diffusion tensor imaging (DTI) [109], and blood oxygen level-dependent functional MRI (BOLD-fMRI) [93], and they ideally could be implemented for identifying functional hallmarks of NPSLE and allow its diagnosis [11,110]. These methods are not detailed here, as this review rather focuses on data obtained in preclinical research performed on murine NPSLE models.

### 3.2. Brain Imaging and MRL/lpr Mice

In NPSLE preclinical research, MRI remains, unfortunately, rarely used, largely due to the high cost necessary for its implementation. However, some data in relation with anatomical findings have been generated and are summarized below.

• MRI

Atrophied cerebral structures in MRL/lpr mice were identified several years ago using paraformaldehyde (PFA)-fixed MRL/lpr brain [111]. Atrophy was noticeable in the superior colliculus, periaqueductal gray matter, pons, and midbrain. However, as PFA fixation could affect both brain morphology and MRI data [112], the conclusions raised from these studies remain to be confirmed.

**Figure 2:**
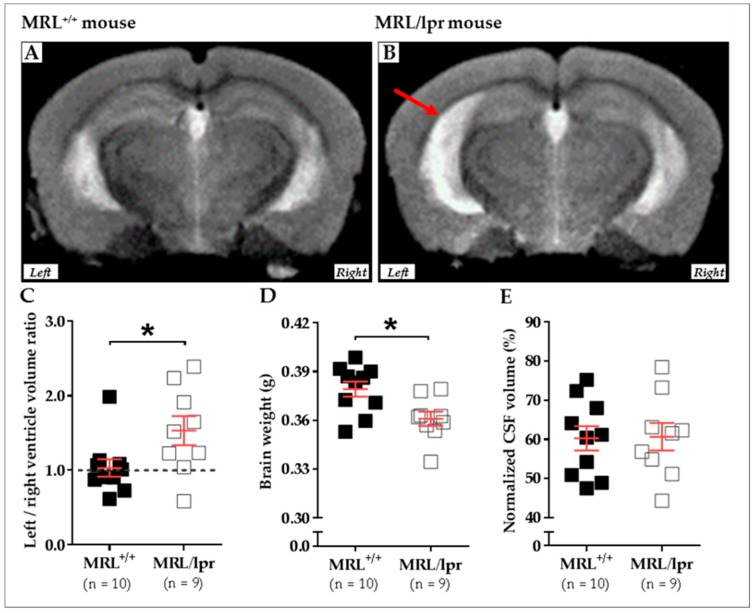
Cerebral abnormalities measured in MRL^+/+^ and MRL/lpr mice. Conventional mid-axial T2-weighted MRI revealed a dilation of ventricles (red arrow) in 16-week-old female MRL/lpr mice (**B**) as compared to the age-matched counterparts MRL^+/+^mice (**A**). In the MRL/lpr strain, this enlargement is more remarkable on the left side, as revealed by analysis of the right/left ventricle volume ratio (**C**). Furthermore, a significant loss of brain weight was detected in these mice (**D**), even if the normalized volume of CSF did not show significant difference between both strains (**E**). Statistics: All data were analyzed with unpaired *t* test, and significance was defined as *p* < 0.05 (*) Errors bars are mean standard deviation. Sample size is indicated as n. Abbreviations: CSF, cerebrospinal fluid; lpr, lymphoproliferation; MRI, magnetic resonance imaging; MRL, Murphy Roths Large.

We recently compared the brain morphology of 16-week-old female MRL^+/+^ (n = 10) and MRL/lpr (n = 9) mice based on transverse relaxation time (T2)-weighted imaging (T2WI) MRI. MRI was performed using a 7/30 Biospec system (Bruker Biospin, Ettlingen, Germany) with ParaVision 6.0.1 software. Transmission was achieved with a quadrature volume resonator (inner diameter of 86 mm), and a standard mouse brain quadrature surface coil (~19 × 19 mm^2^) was used for signal reception (Bruker BioSpin, Ettlingen, Germany). A T2WI axial anatomical dataset was acquired using the RARE sequence (256 × 256 acquisition matrix, 23 slices, 0.5 mm slice thickness, in-plane resolution of 78 × 78 μm^2^, repetition time (TR) of 3 s, echo time (TE) of 30.6 ms, RARE-Factor of 8). Animals were anesthetized with 2% isoflurane, the respiration was noninvasively monitored using a magnetic resonance-compatible system, and the body temperature was maintained constant at 37–38 °C. In this study, an asymmetrical enlargement was noticeable in MRL/lpr brains (Figure 2A,B) with significantly increased ventricular volume on the left side, as demonstrated by the increased left/right ratio for ventricular volume (*p* = 0.035; Figure 2C). Interestingly, a significant loss of brain weight was noted in diseased MRL/lpr mice (*p* = 0.022; Figure 2D), but no difference concerning the CSF volume could be noted between MRL^+/+^ and MRL/lpr mice (*p* = 0.842; Figure 2E). Ventricular dilation and cerebral atrophy have also been previously reported in human NPLSE patients [94]. The meaningful enlargement of the left ventricle in MRL/lpr mice remains largely unstudied but might reveal lateralized brain damage [113] that, for example, has already been observed in neurodegenerative diseases [114]. Such asymmetry might be viewed as a major feature of the disease, which also raises questions on the nature of this lateralization.

• MEMRI

MEMRI is a relatively new imaging technique that is not dependent on blood flow. As a paramagnetic contrast agent, Mn^2+^ is of special interest in neuroimaging, as it enhances MRI contrast in vivo by shorting both T1 (longitudinal) and T2 (transverse) relaxation times. Mn^2+^ ions enter neurons and excitable cells during the excitation phase through voltage-gated calcium channels. As such, MEMRI has unique capabilities for defining cerebral architecture, mapping neuronal pathways, and studying connectivity in morphological and functional imaging studies [115].

This MRI modality was applied to explore the olfactory pathway in mice with experimental NPSLE induced after intracerebroventricular injection of anti-ribosomal-P Abs [116]. Passive transfer of anti-ribosomal-P Abs induced a depressive-like behavior with a significant deficit in olfactory function. MEMRI of these mice demonstrated significant reduction in normalized Mn^2+^ enhancement ratios of olfactory structures, as compared to control mice. Thus, an impaired olfactory neuronal function in mice with experimental depression, mediated by passive transfer of human-anti-ribosomal-P Abs, can be spotted by MEMRI.

• ^1^H-MRS

MRS uses the principles of MRI and, as with nuclear magnetic resonance spectroscopy, allows identification of specific metabolites in vivo at concentrations ranging from 0.5 to 10 mM. ^1^H-MRS determines the ^1^H spectra of molecules [117]. The molecules that can be studied include choline-containing compounds (markers of cellular membrane turnover), creatine (involved in energy metabolism), N-acetylaspartate (NAA; marker of neuronal viability), and lactate (marker of anaerobic metabolism, necrosis, and/or infections). ^1^H-MRS is more sensitive than MRI, evidencing lesions in white and gray matter that appear normal in a conventional MRI of SLE patients.

^1^H-MRS studies have shown significant metabolic differences between MRL^+/+^ and lupus-prone mice in the hippocampus and the cortex [66], structures that, as we already emphasize in this review, are involved in the regulation of cognition and memory. In MRL/lpr mice, these metabolic abnormalities were shown to reflect dysfunction of neuronal (and/or glial) activity, but they did not correlate to structural changes that were detectable by MRI [66]. Both methods are, therefore, necessary to generate a complete picture.

MRI data evidencing an altered cerebral metabolic state in MRL/lpr mice confirm insights on the neuroinflammatory mechanisms of NPSLE in these mice. One major advantage of MRI is its applicability in vivo, allowing the real-time study of the disease progression. At this stage, additional investigations have to be carried out to understand the reasons for the ventricular system asymmetry in MRL/lpr mice, a phenomenon never described until now.

## 4. Neuroinflammation in Lupus-Prone Mice

### 4.1. Characteristic Elements Occurring in the Brain of Lupus-Prone Mice

A summary of the different autoAbs and cytokines detected in MRL/lpr mice and suspected to play a role in NPSLE is shown in Table 2.

#### 4.1.1. Pathogenic AutoAbs

Evidence supporting the role of autoAbs in the CNS pathology and subsequent negative behavioral outcomes includes the higher levels of autoAbs in the serum of diseased MRL/lpr mice that develops earlier in females [57,66] (for extensive reviews, see [36,41]).

There is further demonstration that some of these serum autoAbs recognize brain antigens, such as a subtype of NMDAR glutamate [118,119], an excitatory neurotransmitter. When injected directly into the brain of healthy mice, or when injected peripherally into animals with a breached BBB, these autoAbs are neurotoxic and induce deficits in cognition and emotional behavior [46,120]. We focus attention on this issue in greater detail in Section 4.3.

Intrathecal administration of anti-ribosomal P Abs induces depression-like behavior in the forced swim test [87]. The relation between serum or CSF levels of autoAbs and NPSLE disease processes is complex, but it is likely that intrathecal CSF brain-reactive autoAbs titers [39] may be more critically related to NPSLE pathogenesis and symptoms than serum ones [121].

#### 4.1.2. Cytokines

As it is not necessary that they pass the BBB to regulate neural function, cytokines and chemokines are critical early factors involved in behavioral defects [151]. The role of several cytokines in behavioral disturbances in MRL/lpr mice is supported by numerous studies (see [36]). More specifically, the early dysregulation of cytokine production, especially IL-1, IL-2, IL-6, and tumor necrosis factor (TNF)α, corresponds to the onset of symptoms of depressive-like behavior, such as anhedonia and behavioral despair. Interestingly, anhedonia is ameliorated by cyclophosphamide, which suppresses the typically early and significant rise of IL-6, and can be replicated by exogenous IL-6 [58]. Furthermore, high levels of proinflammatory cytokines may also impair the function of the BBB and, thus, may be permissive to the deleterious effects of certain autoAbs and lymphocytes.

In MRL/lpr mice, the CNS actively responds to the systemic production of several cytokines, as an upregulation of adhesion molecule expression is observed in the brain of diseased mice [129,152]. Upregulated in the peripheral blood of MRL/lpr mice, TNFα, IL-1α, and IL-1β are able to diffuse into the CSF and brain parenchyma [153,154] where they increase the expression of ICAM-1 and VCAM-1, leading to the recruitment of immune cells and subsequently to damage by either direct cytotoxicity or exacerbation of neuroinflammation. As suggested by the observation that inhibition of ICAM-1 in MRL/lpr mice prevents peripheral nerve damage, inhibiting cell adhesion molecule expression in NPSLE could be a promising therapeutic option. However, this line of treatment did not reduce infiltration to the choroid plexus (CP) [155], indicating that, though potentially beneficial, this therapy alone is not sufficient to allow complete resolution of NPSLE signs.

Of the various cytokines suspected to be involved in NPSLE pathogenesis, we make special mention the possible role of macrophage colony-stimulating factor (MIF), which is a proinflammatory cytokine displaying multifunctional properties. Operative in innate and adapted immunity [156,157], it plays an important role in regulating macrophage effector functions and T cell division [158]. MIF has moreover been highlighted in different structures of the CNS (e.g., hippocampus, cortex, cerebellum, pons) [159,160]. Its implication in the pathogenesis of many autoimmune/inflammatory diseases (e.g., multiple sclerosis (MS), Guillain Barré syndrome) is quite well described both in experimental and clinical studies [161,162,163]. MIF contributes also, with sex-specific regulation, to the emergence of depression and psychiatric disorders, likely via the dysregulation of the hypothalamic–pituitary–adrenal (HPA) axis and glucocorticoid secretion [164,165]. MIF is upregulated in SLE patients, where its level correlates positively with disease progression [166], as well as in MRL/lpr mice [146]. Moreover, the deletion of the MIF gene protects MRL/lpr mice from renal and skin manifestations of the disease [167] and reduces depressive symptoms in C57BL/6 mice [164]. Even more, as specific inhibitors of MIF attenuate the clinical course of SLE, therapeutic antagonism of MIF may be investigated as an opportunity for targeted therapy [167]. All these data, as well as the fact that MIF is also involved in autophagy [166], emphasize the important role played by MIF in the effector pathways of immune-mediated inflammatory damage both in SLE patients and murine lupus models [146,166,168,169,170]. They also point to its potential therapeutic use for treating NPSLE. In agreement with this hypothesis, an immunomodulatory peptide, hCDR1, which reduces NPSLE-like symptoms in lupus-prone mice [171], has been found to act on the MIF pathway by reducing its overexpression in the hippocampus [172]. Unfortunately, the effects of MIF and its inhibitors have not yet been further investigated in human NPSLE.

#### 4.1.3. Peripheral Immune Cell Infiltration

Some years ago, the discovery of lymphatic vessels within the dura mater surrounding the brain provoked the dismissal of the existence of CNS immune privilege [173,174,175]. Regarding SLE and NPSLE, the access of peripheral immune cells, of which the best studied are lymphocytes, to the CNS through the CP has now been demonstrated in MRL/lpr mice [22]. Indeed, CD3^+^ T cells are detected in various areas of the MRL/lpr brain (e.g., ventricles, CP, interhemispheric fissure, hippocampus, meninges, stria medullaris, cerebellar parenchyma), while CD19^+^ B cells are only found in the CP and the interhemispheric fissure [176]. Among the CD3^+^ T cells, brain parenchyma is largely infiltrated by inactive CD8^+^ T cells [177], while, in the CP, the larger fraction of infiltrating cells are effector CD4^+^ T cells, which were further identified as T follicular helper (Tfh) cells [73]. Whereas a specific Tfh subset promotes B cell differentiation in the CP, CD4^+^ cells are probably responsible for the increased production of proinflammatory cytokines upon detection of brain-derived self-antigens, leading to the recruitment of other immune cells and parenchymal infiltration [178]. Deletion of the CD4^+^ T cell phenotype has proven to be a successful therapy in mice developing CNS disease, indicating that targeting this cell phenotype might be an approach for treating NPSLE [179]. Unfortunately, this therapeutical option induces deleterious effects in other organs, probably as a result of the deletion of CD4^+^ T regulatory cells, a cell phenotype that attenuates inflammation [180].

#### 4.1.4. Glial Cells

Although poorly explored and not well understood, the role of glial cells, the “sentinels of the brain”, is expected to be central during the neuroinflammatory process of lupus [84]. Some authors reported the upregulation of ionized calcium binding adaptor molecule 1 (Iba1; a microglia/macrophage-specific calcium-binding protein) and of CD68 (glycoprotein expressed by macrophages) in microglia of lupus-prone mice [43,44]. When activated, these cells express proinflammatory molecules and, as mentioned below, perform “synaptic pruning” [43]. Moreover, they probably incur damage by yet unidentified mechanisms. On the other hand, astrocytes are also activated during the disease, as evidenced by elevated astrogliosis in MRL/lpr mouse brain [181]. Further investigations focusing on the function of these cells in the pathogenic mechanisms of NPSLE are needed to better understand their precise role.

### 4.2. Hippocampus as the Possible Primary Target of Neuroinflammatory Lupus

NP symptoms observed in mouse models of lupus are commonly attributed to the dysfunction of the hippocampus, an observation also seen in human patients in whom reduced volumes of the hippocampal CA1 and CA4 regions have been associated with worse cognitive performances [182]. Accordingly, hippocampal neurons in MRL/lpr mice show signs of degeneration when stained by Fluoro-Jade B [153] that are backed by increased levels of proinflammatory cytokines (e.g., IFN-γ, IL-1β, IL-6). Nonetheless, the concomitant presence of anti-inflammatory and immunomodulatory cytokines, for example, IL-10, highlights the complexity of the inflammatory context that occurs in the brains of these mice. In a comparable line, the simultaneous dysregulated production of both proinflammatory and anti-inflammatory cytokines has also been observed in SLE patients at the periphery [183]. This apparently common immunopathogenic aspect strengthens even more the immunopathogenic relevance of the murine models of lupus used to approach human SLE.

Several important properties of the hippocampus may explain both the neuroinflammatory and neurodegenerative processes affecting this cerebral structure during lupus disease. Firstly, the hippocampus is located next to the CP that, in addition to the meninges, as already pointed out above, is a common site of immune peripheral infiltration [176]. Furthermore, immunohistochemistry techniques have revealed that some peripheral immune cells infiltrate the hippocampus itself, homing in on structures that are much beyond their initial site of penetration. Thus, hippocampal proximity to immune infiltration sites could partially explain the susceptibility to NPSLE in this setting. Secondly, the hippocampus is a zone of prominent neurogenesis containing proliferating and maturing cell populations [184,185,186]. In MRL/lpr mice, replicating neuronal cells may display abnormal distribution throughout the CNS [187]. Moreover, the CSF of diseased mice evidences cytotoxicity toward proliferating neuronal cells [121,188]. Two general lines of hypotheses might explain why neurogenesis renders the hippocampus particularly sensitive to autoimmune diseases. One assumption is that newly forming neurons express surface markers different from those of differentiated neurons. Maturing neurons would express NMDAR [189] that is targeted by brain-reactive autoAbs. When it occurs, such binding elicits immediate excitotoxic neuronal death [190]. The second line of assumption questions the involvement of complement factors in neurogenesis and disease pathogenesis [191,192]. The complement components C1q and C3, produced by microglia, can be considered proinflammatory factors, as they play effector roles in a range of functions, including T cell activation and survival, chemotaxis, mast cell degranulation, and macrophage activation. Some of these activities participate in the synaptic loss and death of developing neurons [43,193]. In addition to neurogenesis, the hippocampus is a site of intense synaptic plasticity during ontogenesis and the learning phase [194]. Synaptic plasticity requires the trimming of specific neuronal circuits and reinforcement of others. Normally, this trimming, called “synaptic pruning”, occurs during early fetal development and adolescence in humans (corresponding to 4 weeks post-natally in mice), but it can also be observed during adulthood [195]. Synaptic pruning is performed by microglia in the CNS [196] and also depends on complement factors [194]. Its overactivation results in injury in NPSLE [43]. Although further research is needed, this observation suggests that some zones of higher synaptic plasticity where new neuronal circuits are developing, for instance, in the hippocampus [195], could be particularly sensitive to autoimmune dysfunction. Overall, treatments promoting hippocampal neurogenesis and inhibiting the complement cascade might be regarded as promising therapeutic approaches against NPSLE [193,197,198].

Though the hippocampus seems to be centrally involved in NPSLE, other cerebral areas are affected in this disease, such as the cortex [199], the paraventricular nucleus (PVN) [39,193,200,201], the cerebellum [130], and probably a few others. For example, damage to the PVN alters the sucrose preference behavior [58,60] and increases anxiety. PVN damages are also linked to systemic inflammation as excessive proinflammatory cytokines inhibit the negative feedback of the HPA axis, increase the permeability of the BBB, and disturb the glutamatergic balance [200,202]. At this stage, however, the mechanisms underlying these processes are not clear.

### 4.3. Potential Mechanisms of Neuroinflammation in NPSLE

**Figure 3:**
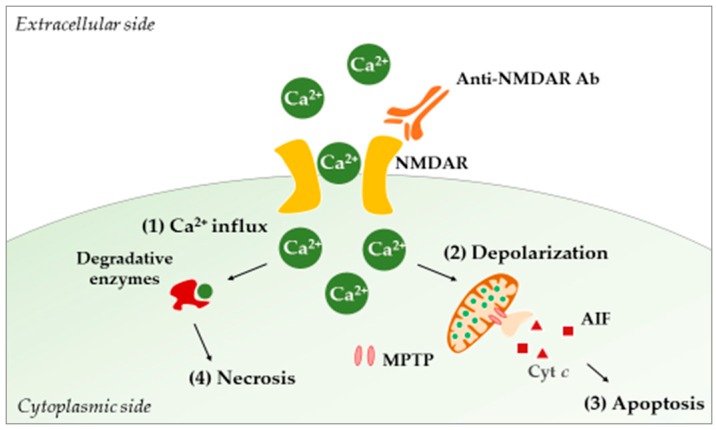
Acute excitotoxicity in NPSLE. The binding of anti-NMDAR Abs to NMDAR allows the free entry of calcium ions (**1**). Intracellularly, the ions are taken up by mitochondria in order to buffer incoming calcium, leading to increased cellular respiration and ROS production. Concomitant with the increase in calcium concentration, the mitochondrial membrane potential collapses, and MPTPs open (**2**). Consequently, proapoptotic molecules (e.g., Cyt *c*, AIF) are released, and apoptosis (controlled neuronal death) occurs (**3**). On the other hand, calcium can activate cytosolic enzymes (e.g., phospholipases, proteases, endonucleases) that will damage neurons intracellularly, leading to necrosis (**4**). Abbreviations: Ab, antibody; AIF, apoptosis-inducing factor; Cyt c, cytochrome *c*; MPTP, mitochondrial permeability transition pore; NMDAR, N-methyl-d-aspartate receptor; ROS, reactive oxygen species.

In lupus-prone mice, different studies using TUNEL and Fluoro-Jade staining have reported neurodegeneration and apoptosis phenomena in the hippocampus, the PVN, and the cortex [153,193,201,203,204]. Today, a causal relationship between such damages and CNS exposure to autoAbs is fairly well documented, and the anti-NMDAR autoAbs are of special interest in NPSLE. These Abs specifically bind the NMDAR and lock it in an open position, leading to the uncontrolled entry of calcium ions into the cell. This passage eventually causes neuronal cell death through a mechanism known as “excitotoxicity” [190] (Figure 3).

**Figure 4:**
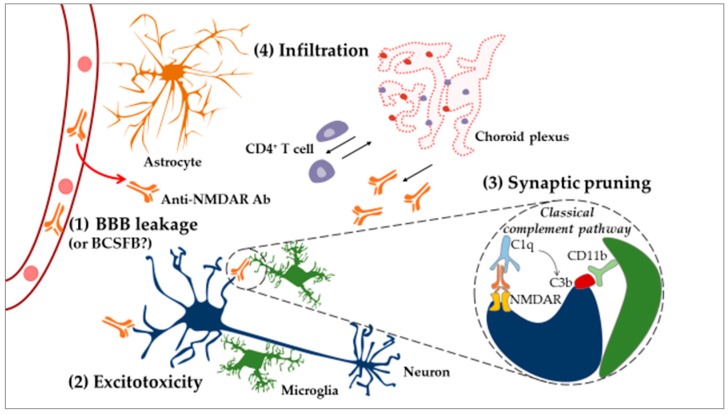
Neuroinflammatory model of NPSLE. In the CNS, under normal physiological conditions, microglial cells act as resident immune cells of the CNS and notably eliminate apoptotic cells. Systemic inflammation renders the BBB permeable to circulating inflammatory factors, for example, certain cytokines and autoAbs (e.g., anti-NMDAR) (**1**). After penetration into the brain, anti-NMDAR Abs can induce apoptotic death of neurons through the process of “excitotoxicity” (**2**). On the other hand, microglial cells can eliminate dendritic spines (the anatomical location of synapses) through a mechanism called “synaptic pruning” (**3**). Hypothetically, anti-neuronal autoAbs bind neuronal antigens recognized by the complement factor C1q (synthetized by the CNS), leading to the production of C3b. Dendritic processes displaying C3b are recognized and phagocytosed by microglia. Furthermore, CNS cells activated following BBB’s leakage upregulate ICAM-1 and VCAM-1 and modulate cytokine expression, resulting in the recruitment of peripheral immune cells (**4**). CD4^+^ T cells infiltrate the CP and the brain parenchyma, while CD19^+^ B cells are only found in the CP. This suggests that CD4^+^ T cells might sample the brain environment in the parenchyma and activate CD19^+^ B cells in the CP, leading to the production of Abs that circulate into the CSF. Abbreviations: Ab, antibody; BBB, blood–brain barrier; BCSFB, blood–cerebrospinal-fluid barrier; CNS, central nervous system; CP, choroid plexus; CSF, cerebrospinal fluid; ICAM-1, intercellular adhesion molecule-1; NMDAR, N-methyl-d-aspartate receptor; VCAM, vascular cell adhesion protein 1.

Of interest is excitotoxicity-related dysfunction, such as calcium overload in mitochondria, as it can result in dendrite degeneration [205]. This process could explain how long-term excitotoxicity may lead not only to neuronal death but also to dendritic spine degeneration and retraction [206] in NPSLE. Neuronal damage can also be induced by these same Abs through the overactivation of the already mentioned mechanism of synaptic pruning [43]. Here, anti-NMDAR autoAbs present on the surface of neurons are recognized by the complement factor C1q [44]. This binding leads to the recruitment of other complement proteins and activation of the classical complement pathway that results in the deposition of the C3b complement factor on the neuronal target surface. In turn, C3b deposits are recognized by the CD11b receptor displayed by microglia. Upon activation of the CD11b receptor, microglia might engulf Ab-tagged structures, removing parts of the neuron [207] (Figure 4).

The mechanism highlighted above is supported by experimental data evidencing that in lupus-like disease, neurons not only die, as noted previously, but also lose their dendritic processes [44,45,204]. The fact that defective synaptic pruning can be stopped by a depletion of microglia or by suppression of C1q [44] or C3 [197] components further corroborates such a mechanism. Interestingly, deletion of the alternative complement pathway could reduce CNS damage [208] but it remains unclear whether the attenuation in brain pathology is related to the reduction in systemic inflammation or if it is brain-specific. It is also unknown whether brain-reactive Abs reach the brain by diffusion through the disrupted BBB [209] or if they are produced intrathecally by infiltrating immune cells that home in on the brain. The presence of plasma cells in the CP suggests that, at least in part, Abs could be produced locally [176].

As indicated above, in MRL/lpr mice, a compromised BBB may allow for the diffusion, into the brain, of molecules present in the systemic circulation [150,210] (Figure 4). It remains to clarify how a leakage in the BBB appears in MRL/lpr mice, however. According to some authors, excessive systemic signaling by circulating cytokines and complement factors could induce a process of controlled cell death (apoptosis) of ECs of the CNS vasculature, thus rendering the BBB leaky [209,211]. In this context, particular reference can be made to the TNF-like weak inducer of apoptosis (TWEAK)/fibroblast growth factor-inducible 14 (Fn14) pathway, which has been identified as the potential culprit of the breach of the BBB [150,181,212]. TWEAK is increased in the cerebral cortices of MRL/lpr mice. Moreover, as compared to MRL/lpr mice, MRL/lpr mice deficient for this pathway perform better in tests of cognition and are less depressed [150]. They also display reduced levels of CSF Igs, decreased peripheral immune cell infiltration, less complement deposition, impaired inducible nitric oxide synthase (iNOS) production, as well as reduced neuronal death [181]. Conversely, intracerebroventricular injection of Fn14 has the opposite effect (e.g., depression-like behavior, learning/memory deficits, macrophage/microglia activation, increased brain cell apoptosis, and astrogliosis) [212]. Consequently, this pathway may be considered as an important target for NPSLE modulation, as it could prevent the initial autoAbs and other effector molecules from penetrating the CNS.

A major question concerning how (and if) brain antigen-specific plasmocytes do mature in NPLSE remains open. An interesting hypothesis involving Tfh cells and generation of ectopic lymphoid structures in the CNS has been proposed and was brought forward with respect to MS [213]. In MS, B cells simultaneously exchange between the CNS and the deep cervical lymph nodes, where they mature into Ab-secreting plasmocytes with the help of local Tfh cells without the need to form ectopic lymphoid structures in the brain [214]. As no ectopic lymphoid structures have been reported in brain parenchyma during NPSLE yet, it is likely that the latter MS mechanism of B cell activation occurs also in NPSLE. It should be noted, however, that Tfh cells are found in the CP of MRL/lpr mice, suggesting that B cells could continue their activation in this structure as well [73].

Even though the precise sequence of neuroinflammatory processes in NPSLE is not fully elucidated, the current results strongly support a determining role of brain-reactive autoAbs. In the CNS, these autoAbs trigger proinflammatory responses by primed CNS cells, which leads to the recruitment of peripheral immune factors and induces further damage (Figure 4).

### 4.4. Is NPSLE a Primarily CNS Disease?

The model of “CNS primarily disease” for NPSLE states that, in MRL/lpr mice, NP events arise due to CNS intrinsic mechanisms. It has been shown that bone marrow transfer from young healthy MRL^+/+^ mice to young (still healthy) MRL/lpr mice could not save the NP phenotype of the recipient mice, leading to the conclusion that systemic inflammation might effectively be dispensable for the emergence of these symptoms [215]. In good agreement with these results, the depletion of B cells in MRL/lpr mice at the age of 14 weeks does not suppress the NPSLE phenotype [216]. Since MRL^+/+^ and MRL/lpr mice differ in one fundamental aspect, which is the presence of a *Fas* gene mutation leading to loss of lymphocyte selection [41], the results obtained by the authors could be explained according to two possible lines of thought: either the *Fas* gene is responsible for the NP symptoms in MRL/lpr mice, or the activated systemic inflammation primes or induces damage in the CNS early in life and persists through life. The first hypothesis would exclude systemic inflammation as the culprit of NP disease.

An opposing comprehensive study showed that most of the behavioral and neurodegenerative symptoms arising in MRL/lpr mice are due to the systemic inflammation and not to the lpr mutation [200]. This study compared an MRL/lpr mouse strain that spontaneously displays an attenuated autoimmune profile and yet still carries the lpr mutation (stock #0006825; Jackson Laboratory) to the original MRL/lpr mouse strain (stock #000485; Jackson Laboratory). This research found that mice with an attenuated disease do not exhibit the vast majority of NPSLE-like symptoms. At last, a study conducted in patients refractory to standard therapy proposed that hematopoietic stem cell transplantation (HSCT) might be proposed as a therapeutic option [217,218]. Interestingly, a remission in NP symptoms has been observed in some cases [219,220], confirming the relation between systemic inflammation and NPSLE symptoms.

Another argument mitigating the hypothesis of “CNS primarily disease” has been evoked with the demonstration that production of type I IFNs, which are produced systemically during the course of the lupus disease [221], prime microglia for damage in lupus-prone mice. Mice deficient for IFN-α/β receptor (IFNAR) are protected from the damaging effects of synaptic pruning [43]. Furthermore, NP symptoms appear early in the life of MRL/lpr mice. For example, depression-like symptoms can be observed at as early as 8 weeks of age in MRL/lpr mice. This was the age that Stock et al. performed the adoptive bone marrow transfer [215]. It is an important point because the autoimmune processes could have had negative effects on the CNS in these mice prior to the bone marrow transfer. Indeed, proinflammatory factors are increased systemically early in the life of MRL/lpr mice [222], and damage by anti-NMDAR Abs lasts even after their removal [45]. Most importantly, it has been shown by two separate studies that the treatment of systemic inflammation attenuates brain damage in lupus-prone mice [223,224]. Another point to consider in this assumption is the effect of maternal autoAbs on the fetus. Some of the behavioral deficits might be difficult to attribute to the systemic inflammation that develops with age, because the fetus could be affected in utero and some specific behavioral deficits could be already present at birth [55,199]. More specifically, it has been shown that, when pregnant mice were immunized against NMDAR or exposed to human anti-NMDAR Abs, their pups developed thinner cortical layers and performed worse in cognitive tasks, showing how the fetus can be affected by the autoimmune status of the mother [199]. In this context, however, it should be noted that the MRL/lpr model, like all experimental models, has its own pitfalls. NPSLE patients could have a genetic CNS intrinsic predisposition to develop NP symptoms, which would require further study on specific gene candidates and additional experiments in an NPSLE-specific mouse model.

In conclusion, even though some behavioral symptoms appear to be related to the *Fas* mutation in MRL/lpr mice, most of these dysfunctions seem to be rather associated with systemic inflammation.

## 5. Therapeutic Approach to NPSLE

### 5.1. Current Treatments

The treatment of NPSLE is currently empirical and symptomatic and remains mainly palliative and nonspecific. Two general therapeutic approaches are distinguished, i.e., anti-ischemic and anti-inflammatory, both respectively stemming from the two presumed mechanisms of NPSLE pathogenesis, i.e., vascular/thrombotic and inflammatory [11].

State-of-the-art therapeutic strategies for NPSLE have been recently reviewed in great detail by Magro-Checa et al. [225] and Hanly et al. [11]. This aspect is not discussed in detail here. Nevertheless, we would like to stress the importance of the prompt management of NP symptoms in SLE patients.

Due to an absence of a “gold standard marker” in NPSLE diagnosis, attribution of NP symptoms to SLE represents a clinical challenge. Without any objective biomarkers, diagnosis solely relies on the exclusion of other potential causes (e.g., infections, hormonal/metabolic dysfunctions, medication-related adverse-effects). In the case of “confirmed” NPSLE diagnosis, therapy should be individualized to each specific patient, and the strategy of choice will depend on major symptoms and their severity. For example, patients presenting with seizures are administered anticonvulsants, those suffering from psychosis are treated with antipsychotics, depression is medicated by antidepressants, and headaches with analgesics or nonsteroidal anti-inflammatory drugs (NSAIDs) [226,227,228]. If symptoms are severe, specific and sometimes aggressive treatments are required [225].

In clinical practice, depending on the suspected underlying pathophysiological processes, therapy is directed at the prevention of ischemic/thrombotic events or at inflammation. Focal symptoms that arise due to the damage or blockage of arteries supplying the brain with oxygen and nutrients are addressed with anti-ischemic therapies, such as anticoagulants and antiplatelets. In the case of diffuse symptoms, which arise due to inflammation, an immunosuppressive therapy is primarily warranted, alone or in combination with anti-inflammatory medication (e.g., corticoids, cyclophosphamide) [225]. The efficacy of cyclophosphamide in treating NPSLE-like disease has been confirmed in MRL/lpr mice [223], thus indicating that novel therapies evaluated in this murine strain might be beneficial in human patients as well (Table 3).

### 5.2. A Potential Therapeutic Option: The Hypothesis of Autophagy

Autophagy (from Ancient Greek, “self-devouring”) is a vital, finely regulated intracellular process that results in the engulfment and destruction of self-components. Its main purpose is the generation of energy during periods of fasting and destruction of faulty cytoplasmic proteins and organelles [232,233,234]. Three major types of autophagy coexist, namely, macroautophagy (the best studied at present and which is negatively regulated by mechanistic target of rapamycin (mTOR)) [235]; microautophagy, which directly engulfs cytosolic material into lysosomes via the formation of characteristic invaginations of the lysosomal membrane [236]; and chaperone-mediated autophagy (CMA), in which an HSPA8/HSC70-containing complex recognizes proteins with a KFERQ-like motif [237].

Increasing evidence suggests autophagy’s important role in both innate and adaptative immunity [238], and thereby its influence on the pathogenesis of inflammatory diseases (see [239]). Autophagy is particularly involved in inflammation by modulating and controlling the development, homeostasis, and survival of inflammatory cells, including B and T lymphocytes. In general, proinflammatory signals and cytokines induce autophagy, while autophagy itself can either increase or reduce proinflammatory cytokine secretion depending on the cellular and inflammatory context (see [240] for an extensive review of the question).

Altered autophagy is found in systemic manifestations of murine SLE [241] and other neuroinflammatory diseases, such as MS and experimental autoimmune encephalomyelitis, an experimental model of MS [85,242,243,244,245]. In these settings, upregulation of the protein kinase mTOR has been described. Indeed, treatment with rapamycin—an immunosuppressant also known as sirolimus—that inhibits mTOR and, thus, stimulates macroautophagy [246], ameliorates some clinical and histological signs of the disease [247,248]. Interestingly, comparable beneficial effects were reported two decades ago in murine SLE [249] and, more recently, also in human SLE [250]. Overall, these data support the view that molecules exerting immunomodulatory effects, and particularly modulating autophagy pathways, should be further investigated and exploited in the therapy of several autoimmune disorders [251].

Autophagy in microglia has been implicated in the development of neuroinflammatory and neurodegenerative diseases [252]. More particularly, a role for microglial autophagy in synaptic pruning and maturation of neuronal connections during development and in the formation of social behaviors has been described recently [253], bringing new insights to NPSLE. Of important note, synaptic pruning in microglia is dependent on microtubule-associated protein light chain 3 (LC3)-associated phagocytosis (LAP) [254]. Dysfunction of LAP is thought to be important in the development of SLE [255], as LAP ablation in the myeloid cell lineage (including microglia) renders mice prone to developing lupus, whereas deletion of macroautophagy itself failed to increase lupus-like symptoms [241]. In the brain of lupus-prone mice, accumulation of neuronal material has been reported in microglia, suggesting that LAP might also be deficient [43]. Concomitantly, elevated levels of autophagy-related proteins (BECLIN-1, LC3, sequestosome-1 (SQSTM1)) have been observed in the brain of diseased MRL/lpr mice, pointing to an increase of autophagy initiation/LAPosome formation with a defect in their degradation rate [256]. On the other hand, and as described above, treatment of SLE with activators of autophagy may lead to better outcomes [249]. The LAP axis is a noncanonical form of autophagy, besides other canonical processes of autophagy, which may be specifically involved in lupus-like disease [241]. To summarize, we hypothesize that NPSLE could greatly benefit from a therapy specifically targeting the formation of LAPosomes in order to increase dendrite arborization and reduce synaptic pruning.

As autophagy can have both beneficial [257] and deleterious [258] effects depending on the disease context [252], we should be very cautious when interpreting the global effects of autophagy-targeting treatment because it could be cell- and time-specific. We already observed that the aberrant autophagy activity (downregulation or hyperactivation) that occurs in pathological settings is not a general feature that equally affects all organs or tissues of any individual [259]. On the contrary, dysregulation can greatly differ from one organ to another, a conclusion also underpinning several autoimmune or non-autoimmune indications [85]. Similarly, it would be fair to emphasize the possible existence of a dichotomic regulation of autophagy (CNS vs. periphery) during SLE. Then, reduction of autophagy should happen at the periphery, which benefits from mTOR inhibitors (e.g., rapamycin), whereas the upregulation of autophagy should occur in the CNS, which would benefit from autophagy inhibitors (e.g., mTOR agonists). If the hypothesis is correct, SLE patients treated with mTOR inhibitors should be very carefully monitored for NPSLE.

We are currently performing behavioral evaluations on MRL/lpr mice administered with a peptide targeting autophagy, particularly CMA, in lupus mice and likely SLE patients also [260,261]. This peptide, called P140, encompasses residues of the U1-70K spliceosomal protein and contains a phosphoserine residue at position 140, hence its name [262]. Our investigations provide promising data for treating SLE patients [263] and possibly also patients with NPSLE: we observed beneficial effects of the peptide on spatial alternation deficits in 17-week-old female MRL/lpr mice [84,85]. Yet, it remains to be determined if P140 can indeed help preserve neuron dendrite arborization in experimental MRL/lpr mice.

## 6. Conclusive Remarks and Perspectives

NPSLE acts as a convincing example of generalized inflammation and autoimmunity leading to CNS damage with behavioral outcomes. However, this is not a unique case of peripheral immune processes propagating at the CNS level and inducing severe disturbances [264]. For example, the assumption that systemic overexpression of IL-6 may contribute to BBB failure and the development of amyotrophic lateral sclerosis has been formulated [265]. It has also been observed that systemic inflammation can induce emotional and cognitive impairments, called sickness behavior, which can resolve on its own. However, in severe cases, like sepsis, the damage can be permanent. This aspect has been extensively reviewed elsewhere [266].

Here, we propose that NPSLE-like disease developing, for example, in lupus-prone MRL/lpr mice, should be regarded as a model of choice for allowing the study of how systemic inflammation affects the brain. Neuroinflammatory mechanisms reported in these mice are comparable to those described in other chronic inflammatory diseases and sepsis. Therefore, they should not be overlooked.

Further research dealing with human NPSLE and murine models of NPSLE should help to better understand the pathophysiological events that trigger and sustain this strong form of lupus disease and allow the validation of useful NPSLE biomarkers. This includes studies based on neuroimaging and investigations aiming to identify serologic and CSF markers. This will be a fundamental step in planning future randomized control trials on the treatment of NPSLE.

## 7. Ethics Statement

Experimental protocols and animal care fulfilled EU regulations (European Council Directive 2013-118) and recommendations of the French national charter for ethics of animal experiments (articles R.214-87 to R.214-126 of the “Code rural et de la pêche maritime”; authorizations no. 04436.02 and no. 3603-2015120211275427v3). The MRI protocol was also approved by the local research institutional animal care and use committee on the ethics of animal experiments (CEEA 35). All efforts were made to minimize animal suffering and respect the 3Rs (Reduce, Refine, Replace).

## Figures and Tables

**Table 1:** ACR case classification of the NP manifestations described in SLE [15].

Central Nervous System		Peripheral Nervous System
**Focal manifestations**	**Diffuse manifestations**	
Cerebrovascular disease	Depression	Cranial neuropathy
Seizures	Cognitive dysfunction	Autonomic neuropathy
Aseptic meningitis	Mood and anxiety disorders ^1^	Mononeuropathy (single/multiplex)
Movement disorder	Psychosis	Polyneuropathy
Myelopathy	Acute confusional state	Plexopathy
Demyelinating syndrome	Headaches ^1^	Myasthenia gravis
		Acute inflammatory demyelinating polyradiculoneuropathy (Guillain-Barré syndrome)

^1^ Anxiety disorders and headaches have been removed in the revised classification suggested by Ainiala et al. (2001) [18]. According to Hanly et al. (2018) [11], posterior reversible encephalopathy syndrome, neuromyelitis optica spectrum disorder, and small fiber neuropathy should be included in a future revision of the ACR classification. Abbreviations: ACR, American College of Rheumatology; NP, neuropsychiatric; SLE, systemic lupus erythematosus. Adapted from Jeltsch-David and Muller (2014) [10].

**Table 2:** Potential NPSLE biological hallmarks found in MRL/lpr mice.

Hallmarks	Location	Levels/Expression	References
**AutoAbs**			
aPL (e.g., anticardiolipin)	Serum	Increased levels	[122]
Anti-dsDNA	Serum	Increased levels	[123]
*Anti-nucleosome* *	*Serum*	*Increased levels*	[124]
Anti-ribosomal P protein	Serum	Increased levels	[125]
*Anti-Sm*	*Serum*	*Increased levels*	[125]
*Anti-ribosomal S10*	*Serum*	*Increased levels*	[126]
Anti-NMDAR	Serum	Increased levels	[57,66]
**Cytokines**			
IL-1β	Serum CNS	Increased levels	[127,128,129,130]
*IL-2*	*T cells*	*Decreased expression*	[131,132]
IL-6	Serum CSF	Increased levels	[127,133,134,135]
*IL-9*	*Serum*	*Increased levels*	[128,130,136]
*IL-10*	*B cells* *CNS*	*Dysregulation*	[128,130,137,138]
*IL-12*	*Serum*	*Increased levels*	[139]
*IL-17*	*Serum*	*Increased levels*	[140]
*IL-18*	*Serum*	*Increased levels*	[141,142]
*IL-21*	*Serum*	*Dysregulation*	[143]
*IL-22*	*Serum*	*Increased levels*	[144]
M-CSF	Serum	Increased levels	[145]
MIF	Serum	Increased levels	[146]
*IFNγ*	*Splenocytes* *CNS*	*Dysregulation*	[128,147,148]
TNFα	Serum	Increased levels	[127,129,149]
TWEAK	CNS	Tendency to increase	[150]
**Receptors**			
*sIL-6R*	*Serum*	*Increased levels*	[133]
Fn14 (TWEAK receptor)	CNS	Increased levels	[150]

* Molecules indicated in italics are those for which relevance in NP manifestations remains to be confirmed. Abbreviations: aPL, antiphospholipid; CSF, cerebrospinal fluid; ds, double-stranded; IFN, interferon; IL, interleukin; lpr, lymphoproliferation; M-CSF, macrophage colony-stimulating factor; MIF, macrophage migration inhibitory factor; MRL, Murphy Roths Large; NMDAR, N-methyl-d-aspartate receptor; sIL-6R, soluble IL-6 receptor; Sm, Smith; TNF, tumor necrosis factor; TWEAK, TNF-like weak inducer of apoptosis.

**Table 3:** Some therapeutic molecules acting in the brain and tested in lupus-prone murine models.

Therapeutic Molecule	Molecular Target	Neuroinflammatory Process	Reference
Crry-Ig	C3 convertase	Complement deposition Apoptosis Neurodegeneration Cytokine production Adhesion molecule expression	[193,197]
Anti-ICAM-1 Ab	ICAM-1	Sciatic nerve conductivity	[155]
Anti-CD4 Ab	CD4^+^ T cells	Immune cell infiltration Inflammation of the CP	[179]
Cyclophosphamide	Immune cell dsDNA	Synaptic pruning	[223]
BI-BTK-1	BTK	Immune cell infiltration	[224]
GW2580	CSF-1R	Cytokine expression	[229]
PLX5622	CSF-1R	Synaptic pruning	[44]
Captopril	ACE	Microglial activation Synaptic pruning	[44]
hCDR1 peptide/Edratide	Anti-dsDNA Ab	Inhibition of neurogenesis Complement deposition Immune cell infiltration Neurodegeneration	[171,198]
FTY720/Fingolimod (*Gilenya*)	S1P receptor	BBB leakage Immune cell infiltration Cytokine production Neurodegeneration	[230,231]
P140 peptide/Lupuzor	HSPA8	CMA regulation	[85]

Abbreviations: Ab, antibody; ACE, angiotensin converting enzyme; BBB, blood–brain barrier; BTK, Bruton’s tyrosine kinase; CMA, chaperone-mediated autophagy; CP, choroid plexus; crry, complement regulator complement receptor 1-related gene/protein-y; CSF-1R, colony stimulating factor 1 receptor; ds, double-stranded; HSPA8, heat shock protein A8; ICAM-1, intercellular adhesion molecule; Ig, immunoglobulin; S1P, sphingosine-1-phosphate.

## Abbreviations

Ab	Antibody
ACR	American College of Rheumatology
aPL	Antiphospholipid
BBB	Blood–brain barrier
BCSFB	Blood–cerebrospinal-fluid barrier
BOLD	Blood oxygen level-dependent
CD	Cluster of differentiation
CMA	Chaperone-mediated autophagy
CNS	Central nervous system
CP	Choroid plexus
CSF	Cerebrospinal fluid
Cyt *c*	Cytochrome *c*
dsDNA	Double stranded DNA
DTI	Diffusion tensor imaging
DWI	Diffusion-weighted imaging
ECs	Endothelial cells
Fas/APO-1/CD95	Apoptosis stimulating fragment
HPA	Hypothalamic–pituitary–adrenal
HSCT	Hematopoietic stem cell transplantation
HSPA8	Heat shock 70-kD protein A8 isoform 1
Iba1	Ionized calcium binding adaptor molecule 1
ICAM-1	Intercellular adhesion molecule 1
IFN	Interferon
IFNAR	IFN-α/β receptor
Ig	Immunoglobulin
IL	Interleukin
iNOS	Inducible nitric oxide synthase
LAP	LC3-associated phagocytosis
LC3	Microtubule-associated protein light chain 3
lpr	Lymphoproliferation
M-CSF	Macrophage colony-stimulating factor
MIF	Macrophage migration inhibitory factor
MPTP	Mitochondrial permeability transition pore
MRA	Magnetic resonance angiography
MRI	Magnetic resonance imaging
MRL	Murphy Roths Large
MRS	Magnetic resonance spectroscopy
MS	Multiple sclerosis
MTI	Magnetic transfer imaging
mTOR	Mechanistic/mammalian target of rapamycin
MTR	Magnetic transfer ratio
NAA	N-acetyl aspartate
NMDAR	N-methyl-D-aspartate receptor
NPSLE	Neuropsychiatric systemic lupus erythematosus
NSAIDs	Non-steroid anti-inflammatory drugs
NZW	New Zealand White
NZB	New Zealand Black
PAF	Paraformaldehyde
RARE	Rapid acquisition with relaxation enhancement
ROS	Reactive oxygen species
SQSTM1/p62	Sequestosome-1/p62
T1	Longitudinal relaxation time
T2	Transverse relaxation time
TE	Echo time
Tfh	T follicular helper
TNF	Tumor necrosis factor
TR	Repetition time
TWEAK	TNF-like weak inducer of apoptosis
VCAM-1	Vascular cell adhesion protein 1
vs.	versus
